# Diabetic macular edema and proliferative diabetic retinopathy treated with anti-vascular endothelial growth factor under the reimbursement policy in Taiwan

**DOI:** 10.1038/s41598-021-04593-x

**Published:** 2022-01-13

**Authors:** Ming-Chieh Hsieh, Chieh-Yin Cheng, Kun-Hsien Li, Chih-Chun Chuang, Jian-Sheng Wu, Sheng-Ta Lee, Wei-Yang Lu, Shin-Lin Chiu, Yu-Ling Liu, San-Ni Chen

**Affiliations:** 1 Department of Ophthalmology, Eye center, China Medical University Hospital, Taichung City, Taiwan; 2 Department of Ophthalmology, Changhua Christian Hospital, Changhua City, Taiwan; 3 College of Nursing and Health Sciences, Da-Yeh University, Changhua City, Taiwan; 4 Department of Ophthalmology, China Medical University, Taichung City, Taiwan; 5 Department of Optometry, Da-Yeh University, Changhua City, Taiwan; 6 School of Medicine, Chung-Shan Medical University, Taichung, Taiwan

**Keywords:** Diabetes, Diabetes complications, Retinal diseases

## Abstract

The purpose of this retrospective interventional case series is to compare the functional and anatomical outcomes in eyes with diabetic macular edema (DME) and proliferative diabetic retinopathy (PDR) treated intravitreally with aflibercept or ranibizumab under the Taiwan National Insurance Bureau reimbursement policy. 84 eyes were collected and all eyes were imaged with spectral-domain optical coherence tomography (SD-OCT), color fundus photographs (CFPs), and fluorescein angiography (FA). At 24 months after therapy initiation, the logMAR BCVA improved from 0.58 ± 0.33 to 0.47 ± 0.38 (*p* < 0.01), the CRT decreased from 423.92 ± 135.84 to 316.36 ± 90.02 (*p* < 0.01), and the number of microaneurysms decreased from 142.14 ± 57.23 to 75.32 ± 43.86 (*p* < 0.01). The mean injection count was 11.74 ± 5.44. There was no intergroup difference in logMAR BCVA (*p* = 0.96), CRT (*p* = 0.69), or injection count (*p* = 0.81). However, the mean number of microaneurysms was marginally reduced (*p* = 0.06) in eyes treated with aflibercept at the end of the follow-up, and the incidence rates of supplementary panretinal photocoagulation (PRP) (*p* = 0.04) and subthreshold micropulse laser (SMPL) therapy sessions (*p* = 0.01) were also reduced. Multivariate analysis revealed that only initial logMAR BCVA influenced the final VA improvements (odds ratio (OR) 0.49, 95% confidence interval (CI) 0.21 ~ 0.93, *p* < 0.01); in contrast, age (OR − 0.38, 95% CI − 6.97 ~ − 1.85, *p* < 0.01) and initial CRT (OR 0.56, 95% CI 0.34 ~ 0.84, *p* < 0.01) both influenced the final CRT reduction at 24 months. To sum up, both aflibercept and ranibizumab are effective in managing DME with PDR in terms of VA, CRT and MA count. Eyes receiving aflibercept required less supplementary PRP and SMPL treatment than those receiving ranibizumab. The initial VA influenced the final VA improvements at 24 months, while age and initial CRT were prognostic predictors of 24-month CRT reduction.

## Introduction

Anti-vascular endothelial growth factor (anti-VEGF) agents have been accepted as the first-line treatment for diabetic macular edema (DME). Recently, protocol S also showed the good efficacy of ranibizumab in regressing preretinal and prepapillary neovascularization and lowering the stage of diabetic retinopathy (DR) as part of the management of proliferative DR (PDR)^1^. In addition, eyes receiving ranibizumab had a reduced chance of developing peripheral visual field loss and center-involved macular edema. Similar study results were shown at the 1-year follow-up in the CLARITY randomized clinical trial using aflibercept^2^.

Anti-VEGF agents, including both ranibizumab and aflibercept, have been reimbursed by the National Taiwan Health Insurance Bureau for DME but not PDR since 2014. A lifetime maximum of 8 injections per eye are eligible for reimbursement, and patients are not allowed to shift from one anti-VEGF agent to another. In this study, we aimed to observe the clinical outcomes of anti-VEGF treatment in eyes with DME and PDR under the reimbursement policy of the National Taiwan Health Insurance Bureau and compare the clinical outcomes of ranibizumab and aflibercept treatment in eyes with DME and coexistent PDR.

## Results

Eighty-four eyes of 72 patients (M:F = 41:31, mean age: 58.57 ± 10.76) were included in the study. Forty-one eyes that received aflibercept were categorized into group 1, and 43 eyes that received ranibizumab were categorized into group 2. The mean logMAR best-corrected visual acuity (BCVA) at baseline was 0.58 ± 0.33. There was no significance intergroup difference in the patients’ basic profiles, initial logMAR BCVA, CRT, or retinal morphology; these data are summarized in Table 1.

**Table 1:** Baseline characteristics.

	Gr 1 (Aflibercept) N = 41	Gr 2 (Ranibizumab) N = 43	*p*
Age	57.44 ± 10.87	59.65 ± 10.82	0.41
Sex (M: F)	18:17	23:14	0.48
HbA1c	7.53 ± 1.33	7.46 ± 1.75	0.85
Eye (OD: OS)	19:22	26:17	0.27
logMAR BCVA	0.60 ± 0.33	0.56 ± 0.33	0.59
CRT	426.02 ± 159.87	423.91 ± 111.28	0.94
VH	8/41	9/43	1.00
Pseudophakia	4/41	4/43	1.00
Previous PRP	11/41	15/43	0.48
Presence of ERM	11/41	19/43	0.12
DRIL	337.44 ± 296.97	311.56 ± 318.24	0.70
MAs	145.93 ± 46.77	137.19 ± 65.25	0.48
NV (disc size)	1.63 ± 1.80	1.28 ± 2.20	0.42

*Gr* group, *M* male, *F* female, *OD* right eye, *OS* left eye, *logMAR BCVA* best-corrected visual acuity in the logarithm of the minimal angle of resolution, *CRT* central retinal thickness, *VH* vitreous hemorrhage, *PRP* panretinal photocoagulation, *ERM* epiretinal membrane, *DRIL* Disorganization of the retinal inner layers, *MAs* microaneurysms, *NV* retinal neovascularization.

At 24 months after the initiation of the treatment, the mean number of injections was 11.74 ± 5.44 (*p* = 0.91), the logMAR BCVA improved from 0.58 ± 0.33 to 0.47 ± 0.38 (*p* < 0.01), the central retinal thickness (CRT) decreased from 423.92 ± 135.84 to 316.36 ± 90.02 (*p* < 0.01), and the counts of microaneurysms (MAs) decreased from 142.14 ± 57.23 to 75.32 ± 43.86 (*p* < 0.01). There was no difference in the injection count, logMAR BCVA or CRT between the two groups (Table 2).

**Table 2:** Outcomes at 24 months after treatment.

	Gr 1 N = 41	Gr 2 N = 43	*p*
IVI (times)	12.02 ± 6.57	11.89 ± 4.86	*0.91*
logMAR BCVA	0.47 ± 0.41	0.47 ± 0.37	0.96
Delta logMAR BCVA	− 0.13 ± 0.37	− 0.09 ± 0.33	0.65
CRT	319.51 ± 133.52	309.67 ± 90.95	0.69
Delta CRT	− 106.51 ± 194.87	− 104.21 ± 114.89	0.95
DRIL	304.02 ± 271.57	295.28 ± 277.09	0.88
New VH	9/41	14/43	0.33
VH that requested PPV	2/41	4/43	0.68
New ERM	3/41	8/43	0.20
ERM	11/41	15/43	0.48
Pseudophakia	15/41	17/43	0.83
Supp. PRP rates	9/41	19/43	0.04
SMPL rates	2/41	11/43	0.01
FRP rates	2/41	5/43	0.43
MAs	64.90 ± 30.81	86.13 ± 53.10	0.06
NV	0.05 ± 0.23	0.14 ± 0.68	0.47
NVI	0/41	1/43	1.00
NVG	1/41	5/43	0.20
Endophthalmitis	0/41	1/43	1.00
RRD	1/41	0/43	0.49
TRD	0/41	1/43	1.00

*IVI* intravitreal injection, *VH* vitreous hemorrhage, *ERM* epiretinal membrane, *supp.* Supplementary, *PRP* panretinal photocoagulation, *SMPL* subthreshold micropulse laser, *logMAR BCVA* best-corrected visual acuity in the logarithm of the minimal angle of resolution, *CRT* central retinal thickness, *DRIL* Disorganization of the retinal inner layers, *NVI* neovascularization of iris, *NVG* neovascular glaucoma, *RRD* rhegmatogenous retinal detachment, *TRD* tractional retinal detachment, *PPV* pars plana vitrectomy, *MAs* microaneurysms, *NV* retinal neovascularization.

On the other hand, for cases that had more than 8 injections, macular edema was the main reason (93.86%), other reasons including vitreous hemorrhage (3.22%), new retinal neovascularization (0.58%), neovascularization of iris (0.58%), and preparing for pars plana vitrectomy (PPV) to eliminate possible hemorrhage during operation (1.17%). There was no difference between cases received less or equal to 8 and cases had additional injections regarding final BCVA, CRT, and MAs.

New-onset vitreous hemorrhage (VH) was noted in 27.38% of eyes. The incidence of new VH during the follow-up period was higher in the ranibizumab group than in the aflibercept group, but the difference was not statistically significant (*p* = 0.33). PPV was performed in 7.14% of eyes for recurrent or non-clearing VH. Although the rate of PPV was higher in eyes receiving ranibizumab, there was no significant difference (4.88% in the aflibercept group and 9.30% in the ranibizumab group, *p* = 0.68).

Supplementary pan-retinal photocoagulation (PRP) was needed in 33.33% of eyes. The supplementary PRP were prescribed in cases whom had new onset VH (71.42%), neovascular glaucoma (14.29%), faint PRP scars based on the physician’s experience (10.71%), and neovascularization of iris (3.57%). More cases in the ranibizumab group than in the aflibercept group required supplementary PRP (*p* = 0.04), and more sessions of supplementary PRP were needed (*p* = 0.02). A comparison was made between PRP naïve and PRP-treated at baseline, but no difference was noticed regarding the supplementary sessions (aflibercept group *p* = 0.46, ranibizumab group *p* = 0.08), and the rate (aflibercept group *p* = 0.68, ranibizumab group *p* = 0.52). Subthreshold macular pulse laser (SMPL) for persistent DME was performed in 15.48% of eyes, with a significantly higher number in the ranibizumab group (*p* = 0.01). In contrast, the rate of focal retinal photocoagulation (FRP) in all eyes was 8.33%, with no significant difference between the two groups (*p* = 0.43). For neovascular glaucoma (NVG) and neovascularization of the iris (NVI), the incidence was 7.14% and 1.19%, respectively, for all eyes, and no differences were noted between these 2 groups. The area of retinal neovascularization within the seven field areas also markedly decreased for all eyes (from 1.30 ± 1.90 to 0.10 ± 0.52, *p* < 0.01), and no difference was noted between the 2 groups (*p* = 0.47). A decreased area of disorganization of Retinal Inner Layers (DRIL) (from 324.19 ± 306.46 to 299.55 ± 272.79, *p* = 0.5) was also noticed; however, the difference was not statistically significant.

Regarding procedure-related complications, 1 eye in group 1 had rhegmatogenous retinal detachment; 1 eye in group 2 had endophthalmitis; and 1 eye in group 2 had tractional retinal detachment, which required PPV.

The multivariate analysis revealed that only initial logMAR BCVA influenced the final VA improvements (OR 0.49, 95% CI 0.21 ~ 0.93, *p* < 0.01); on the other hand, age (OR − 0.38, 95% CI − 6.97 ~ − 1.85, *p* < 0.01) and initial CRT (OR 0.56, 95% CI 0.34 ~ 0.84, *p* < 0.01) both influenced the final CRT reduction at 24 months (**Table ****3**).

**Table 3:** multivariable analysis of better functional and anatomical improvements at 24 months.

	Odds ratio (95% CI, *p*)		
	logMAR BCVA	CRT	MAs
Anti-VEGF	0.08 (− 0.13 ~ 0.24, 0.57)	0.10 (− 0.52 ~ 0.33, 0.34)	− 0.11 (− 36.24 ~ 9.02, 0.23)
Sex	0.05 (− 0.16 ~ 0.22, 0.73)	0.04 (− 40.09 ~ 61.07, 0.68)	0.14 (− 4.44 ~ 40.75, 0.11)
Age	− 0.22 (− 0.02 ~ 0.01, 0.13)	− 0.38 (− 6.97 ~ − 1.85, < 0.01)	− 0.11 (− 1.72 ~ 0.48, 0.27)
HbA1c	− 0.04 (− 0.06 ~ 0.05, 0.76)	− 0.10 (− 23.03 ~ 7.94, 0.33)	− 0.03 (− 7.97 ~ 5.58, 0.72)
IVI (times)	− 0.09 (− 0.02 ~ 0.01, 0.50)	− 0.02 (− 5.37 ~ 4.467, 0.86)	0.04 (− 1.71 ~ 2.74, 0.65)
Initial logMAR BCVA	0.49 (0.21 ~ 0.93, < 0.01)	0.15 (− 35.86 ~ 156.128, 0.22)	− 0.09 (− 62.30 ~ 26.08, 0.42)
Initial CRT	− 0.07 (− 0.01 ~ 0.01, 0.67)	0.56 (0.34 ~ 0.84, < 0.01)	0.06 (− 0.08 ~ 0.15, 0.55)
Supp. PRP	− 0.06 (− 0.08 ~ 0.05, 0.65)	− 0.14 (− 29.48 ~ 6.69, 0.21)	0.08 (− 5.11 ~ 11.70, 0.44)
SMPL	− 0.04 (− 0.14 ~ 0.11, 0.78)	< − 0.01 (− 29.05 ~ 28.55, 0.99)	− 0.07 (− 21.63 ~ 8.84, 0.40)
Initial MAs	− 0.17 (− 0.01 ~ 0.01, 0.19)	− 0.04 (− 0.52 ~ − 0.33, 0.67)	0.71 (0.59 ~ 0.97, < 0.01)

*logMAR BCVA* best-corrected visual acuity in logarithm of minimal angle of resolution, *anti-VEGF* anti-vascular endothelial growth factor, *IVI* intravitreal injection, *CRT* central retinal thickness, *Supp.* Supplementary, *PRP* panretinal photocoagulation, *SMPL* subthreshold micropulse laser, *MAs* microaneurysms.

## Discussion

Recently, there has been increasing interest in the use of anti-VEGF agents to treat PDR^1^. Both ranibizumab and aflibercept have been shown to prevent DR progression and improve scores on the Diabetic Retinopathy Severity Scale (DRSS)^1–5^.

In this retrospective study, patients with DME and PDR had significant visual improvement, CRT reduction, and decreased MA counts after 24 months of anti-VEGF treatment. MA counts have been well characterized in a previous study as a biomarker for grading DR severity^6^. MA counts have recently been shown to improve after intravitreal injection of anti-VEGF^7–9^, which suggests an improvement in DR. Although the total number of injections is limited by the reimbursement policy of the Taiwan Insurance Bureau, good anatomical and functional outcomes can be achieved.

On the other hand, the length of DRIL did not change in the present study (*p* = 0.51). Nicholson et al. reported the association between DRIL and capillary nonperfusion on FA^10^. A recent study further demonstrated the presence of multilayer ischemia in DRIL by OCT angiography^11^. The stationary length of DRIL may imply that the ischemic process of DR remains undeteriorated during the treatment period, which echoes the finding of another study that the area of nonperfusion did not change despite 3 monthly injections of anti-VEGF^8^.

PPV for the complications of PDR was carried out in 6/84 patients (7.14%) in our series. Compared to the previous study of protocol S, which compared the efficacy of PRP and intravitreal injection (IVI) of ranibizumab in controlling PDR^1^, the incidence of PPV in our study was lower than that in the PRP group but higher than that in the IVI group in protocol S. However, since the number of injections per patient was far lower in our study than in protocol S, it is not surprising that there was a higher incidence of PPV.

In this study, although there was no intergroup difference in BCVA or CRT at 24 months, MAs were marginally higher in the ranibizumab group. In addition, supplementary PRP was performed significantly more in the ranibizumab group than in the aflibercept group (*p* = 0.04). These results may implicate that aflibercept improved the grade of DR more effectively than ranibizumab, as shown in previous reports^12,13^. Since the placental growth factor (PGF) correlates with the severity of PDR^14^, the different molecular mechanisms between aflibercept and ranibizumab might be one of the causative reasons^15^. In addition, there was a significantly higher chance of eyes in the ranibizumab group receiving SMPL (*p* = 0.01) to further control the status of macular edema.

However, in the multivariate analysis, when we considered each patient’s basic profile, the type of anti-VEGF the patient received, the number of injections, the initial VA, the CRT, the MA count, and the use of laser therapy, only the initial VA predicted the VA improvements after 24 months of treatment (OR 0.49, *p* < 0.01): the worse the initial VA, the more it would improve by 24 months. Similar results were noticed for the CRT reduction: the greater the initial CRT, the more it would decrease. In addition, age was another factor influencing CRT reduction. These results were similar to previous reports that younger patients with greater initial CRT were likely to have greater CRT reduction^12,16^.

There was no detected difference in ocular safety concerns between aflibercept and ranibizumab during the follow-up period. The rates of endophthalmitis and other injection-related adverse events were low, as in previous studies [1 ~ 5, 12]. The rates of NVI, NVG, and tractional retinal detachment were also low and showed no difference between the two groups.

Previous studies have tried to reflect the clinical practice in DME treated with anti-VEGF in Taiwan, but the follow-up period was relatively shorter^17–19^. In addition, no study has presenting the relationship between anti-VEGF and cases coexist PDR yet. To our best knowledge, this is the first real-world data demonstrating such cases treated with anti-VEGF in Taiwan. However, this study had several limitations. The data were collected retrospectively, and in place of strict protocols, doctors and patients decided at their own discretion whether procedures would be performed. Moreover, in Taiwan, only a limited number of anti-VEGF injections can be reimbursed, which may affect patients’ willingness to have additional IVIs beyond the reimbursed injections. Future prospective studies with larger sample sizes and long-term follow-up are necessary to clarify the benefit of anti-VEGF agents and the difference between different anti-VEGF agents in the treatment of PDR and DME.

In conclusion, anti-VEGF agents including ranibizumab and aflibercept were effective in improving visual acuity and reducing CRT in eyes with DME and PDR under the reimbursement policy of the Taiwan National Insurance Bureau. Although there was no difference in BCVA and CRT outcomes between ranibizumab and aflibercept at 2 years after treatment, the numbers of MAs, supplementary PRP sessions, and SMPL sessions were higher in the ranibizumab group than in the aflibercept group. However, only the initial VA influenced the VA improvements at 2 years.

## Methods

The study was a retrospective, interventional, comparative study of eyes with DME and PDR that were reimbursed for either ranibizumab or aflibercept from the National Insurance Bureau for DME treatment at Changhua Christian Medical Center, a tertiary center in Taiwan, from November 2014 to March 2020. The study was approved by the Institutional Review Board of Changhua Christian Hospital and was conducted according to the tenets of the Declaration of Helsinki. Informed consent was obtained from all patients.

All the eyes included in this study fulfilled the criteria for reimbursement for DME by the National Taiwan Insurance Bureau, which included the following: 1. presence of DME, 2. CRT greater than 300 microns as measured by spectral domain optical coherence tomography (SD-OCT), 3. Best-corrected decimal visual acuity ranging from 0.05 to 0.5, and 4. HbA1c below 10%.

In addition to the reimbursement criteria, we applied additional inclusion criteria as follows: 1. age 18 to 80 years, 2. available standard 7-field color fundus photography (CFP), 3. PDR according to fluorescein angiography (FA), 4. no VH obscuring the posterior pole at baseline so that that macular morphological status in the central 6 mm could be obtained by SD-OCT (Spectralis, Heidelberg Engineering, Heidelberg, Germany), 4. treatment with either aflibercept or ranibizumab, without switching to other agents, and 5. more than 2 years of follow-up.

Each patient’s age, sex, and HbA1c level were recorded. BCVA, slit-lamp biomicroscopy findings, indirect ophthalmoscopy results and SD-OCT data from each visit were documented. The procedures and surgeries received during each clinic visit were documented as well.

CFP and FA images were assessed for MAs, retinal neovascularization area, and nonperfusion within the standard seven fields. MAs were quantified in FA and CFP by two independent physicians (Hsieh MC and Cheng CY) using Image J. In the event of disagreement, a third investigator (Chen SN) was consulted for final determination. The MAs were defined as small circular hyperfluorescence objects with surrounding choroidal background in early phase FA^20^.

The CRT was measured by SD-OCT at a diameter of 100 microns centered at the fovea. DRIL was defined as any unidentifiable boundaries between the ganglion cell-inner plexiform layer complex, inner nuclear layer, and outer plexiform layer^21,22^. The length of DRIL was measured at a 6 mm scan centered on the fovea.

### Outcomes

Outcome measures included the final BCVA, mean changes in BCVA from baseline to 2 years, CRT, the regression rate of neovascularization, numbers of injections, sessions of supplementary PRP, FRP, SMPL, presence of VH, NVI, NVG, new-onset epiretinal membrane, and the numbers of MAs within zone 1 and the seven fields. The number of cases that underwent vitrectomy because of tractional retinal detachment and persistent or frequent recurrence of VH was also documented. Other related condition including endophthalmitis, cataract surgery, and retinal detachment were also recorded.

### Statistical analysis

Decimal visual acuity was converted to logMAR units for statistical analysis. All analyses were performed using SPSS 23.0 software (SPSS, Chicago, IL, USA). The Mann–Whitney U test and the Wilcoxon signed-rank test were used for continuous variables. The chi-square test or Fisher’s exact test was used for categorical variables. Factors influencing the outcome were analyzed by logistic regression. The odds ratio was calculated, and the 95th percentile confidence intervals (95% CI) were determined. A p value less than 0.05 was considered statistically significant.
